# 138. Tele-COVID Rounds and Tele-Stewardship Surveillance Reduces Antibiotic Use in COVID-19 Patients Admitted to 17 Small Community Hospitals

**DOI:** 10.1093/ofid/ofab466.138

**Published:** 2021-12-04

**Authors:** John J Veillette, Stephanie C Shealy, Stephanie Gelman, Edward A Stenehjem, Steven K Throneberry, Michael Pirozzi, Brandon J Webb, Dustin Waters, Valoree K Stanfield, Nancy A Grisel, Todd J Vento

**Affiliations:** 1 Intermountain Healthcare, Murray, UT; 2 McKay-Dee Hospital - Intermountain Healthcare, West Haven, Utah

## Abstract

**Background:**

Early bacterial co-infection is rare in hospitalized COVID-19 patients, yet antibiotics are commonly prescribed. Antibiotic stewardship (AS) intervention is needed, especially in small community hospitals (SCHs), which often lack access to AS expertise.

**Methods:**

We implemented daily remote multidisciplinary tele-COVID rounds (synchronous case review between SCH providers and ID clinicians) and tele-stewardship surveillance (ID pharmacist review of COVID patients on antibiotics) on 6/24/2020 in 17 SCHs. We retrospectively included adult symptomatic COVID-19 admissions between 3/2020 and 4/2021. The primary outcome was early use of antibiotics for pneumonia (started within 48 hours of admission); mean monthly days of therapy per 1,000 patient days (DOT) were compared pre- (3/2020-6/2020) and post-intervention (7/2020-4/2021). Secondary outcomes were early use of antibiotics for any indication, estimated days of antibiotics avoided (comparing pre- and post-intervention DOT), and in-hospital mortality. Analyses were conducted using a two-tailed unpaired t-test (antibiotic use) or Fisher’s exact test (mortality).

**Results:**

Of the 1,976 patients included (124 pre- vs. 1852 post-intervention), 55.4% were male and 85.5% were white. Patients in the pre-intervention group were more likely to require hospital transfer [21.8% vs 8.8% (p< 0.001)] and ICU admission [18.5% vs. 9.7% (p=0.003)]. We observed a significant decrease in mean use of early antibiotics for pneumonia [656.9 vs. 240.1 DOT (p< 0.001)], including among non-ICU patients only [603.6 vs 240.2 DOT (p< 0.001)]. Early antibiotic use for any indication also decreased [686.2 vs. 359.3 DOT (p< 0.001)]. An estimated 3,697 days of unnecessary antibiotics for pneumonia were avoided in the 10-months post-intervention [370 days per month (95% CI 304 – 435)]. Unadjusted in-hospital mortality was not different pre- vs post-intervention (0.8% vs. 2.0%, p=0.511), but was higher among those prescribed early antibiotics (4.4% vs 0.5%, p< 0.001).

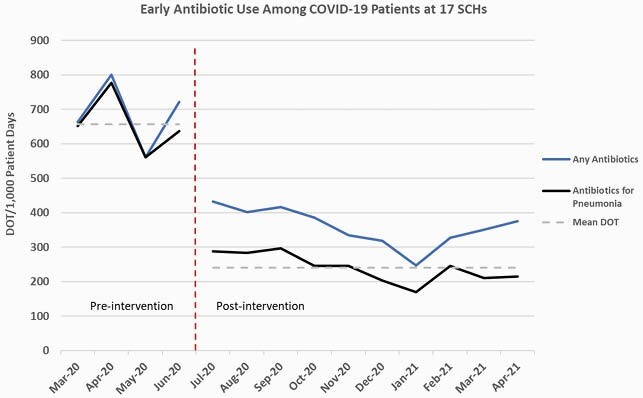

**Conclusion:**

A significant, sustained reduction in antibiotic use among COVID-19 patients at 17 SCHs was observed after implementation of tele-COVID rounds and tele-stewardship surveillance without an observed difference in mortality.

**Disclosures:**

**All Authors**: No reported disclosures

